# The combined effects of yogurt and exercise in healthy adults: Implications for biomarkers of depression and cardiovascular diseases

**DOI:** 10.1002/fsn3.772

**Published:** 2018-09-07

**Authors:** Hyun‐Kyung Kim, Soo‐Hwan Kim, Chul‐Soo Jang, Sung‐In Kim, Chang‐Oh Kweon, Byung‐Won Kim, Jae‐Ki Ryu

**Affiliations:** ^1^ Department of Biomedical Laboratory Science College of Nursing and Health Science Gimcheon University Gimcheon‐si South Korea; ^2^ Department of Integrated Biomedical and Life Science Graduate School Korea University Sungbuk‐Gu South Korea

**Keywords:** cardiovascular disease, depression, exercise, high‐sensitivity c‐reactive protein, serotonin, yogurt

## Abstract

Several studies have reported individual benefits of yogurt and exercise on health; however, their combined effects remain unclear. Twenty‐four healthy individuals participated in the study and were randomly assigned to the following four groups: control, yogurt, exercise, and combination. The participants consumed yogurt and exercised for 2 weeks, and we examined the combined effects of yogurt and exercise on physiological biomarkers. Individually, yogurt and exercise did not exert a significant effect on biomarkers of depression or cardiovascular disease, although vitamin D levels increased in the exercise group. However, in the combination group, serotonin levels increased, while levels of triglycerides and high‐sensitivity C‐reactive protein, which are biomarkers for cardiovascular diseases, decreased. In conclusion, the results of the study showed that, in healthy individuals, a combination of yogurt and exercise led to greater increases in serotonin levels and reductions in triglyceride and high‐sensitivity C‐reactive protein levels, relative to those observed for yogurt or exercise alone; therefore, this combination could have implications for the prevention of depression and cardiovascular disease.

## 1 INTRODUCTION

Yogurt, which contains probiotic microbes, has been known to exert beneficial effects on physiological conditions such as cardiovascular diseases (CVDs) and depression (Astrup, 2014; Huang, Wang, & Hu, 2016). In a meta‐analysis examining depression, probiotic consumption led to significant reductions in depression scale scores in both healthy control participants and patients with depression (Huang et al., 2016), and low‐density lipoprotein cholesterol (LDL‐C) levels decreased with yogurt consumption (Astrup, 2014). Exercise serves as a physiological stressor, and sustained exercise has been known to relieve depressive symptoms and CVD. A recent study reported that exercise exerted an antidepressant effect and decreased the risk of CVD by reducing triglyceride (TG) levels (Hearing et al., 2016; Villella & Villella, 2014).

Serotonin is a monoamine neurotransmitter derived from tryptophan (Richard et al., 2009) and has been shown to regulate mood and mood‐related disorders associated with low serotonin levels. Vitamin D is a fat‐soluble, natural secosteroid that regulates calcium and phosphorus absorption in the intestine. Anglin, Samaan, Walter, and McDonald (2013) reported that low vitamin D levels were related to depression. Therefore, vitamin D, in addition to serotonin, could be a biomarker for depression.

The main risk factors for CVD include an abnormal lipid profile; increase in total cholesterol, LDL‐C, and TG levels; and reductions in high‐density lipoprotein cholesterol (HDL‐C) levels (Grundy, Pasternak, Greenland, Smith, & Fuster, 1999). Oxidized LDL‐C and TGs are known to trigger the production of foamy macrophages, which play an important role in the progression of atherosclerosis (Itabe, Obama, & Kato, 2011; Rohilla, Dagar, Rohilla, Dahiya, & Kushnoor, 2012; Talayero & Sacks, 2011), and atherosclerosis can lead to CVD via the rupture of plaques (Frostegård, 2013). Therefore, a balanced lipid profile is very important for the prevention of CVD. Moreover, hs‐CRP is a sensitivity immunoassay that is used to measure low CRP levels in healthy individuals (Koh et al., 2005) and has been identified as a biomarker for CVD risk and inflammation (Soriano‐Guillén et al., 2008).

Depression is defined as a mental health disorder that negatively affects mood and overall physical well‐being (Vilagut, Forero, Barbaglia, & Alonso, 2016). People with depression could find performing physical activity, concentrating, and eating difficult and might exhibit suicidal ideation (Kim, Cho, Park, & Park, 2015). More than 300 million people are estimated to experience depressive disorders, and this number appears to be increasing (Solem et al., 2017). Similar to depression, CVD is a common condition and a main cause of global mortality (Nelson, 2013). Therefore, the prevention and management of depression and CVD are important.

Previous research has demonstrated individual effects of yogurt and exercise on depression and CVD; however, their combined effects remain unclear. Therefore, we sought to determine whether the combination of yogurt and exercise exerted preventive effects on depression and CVD. To this end, we assessed changes in biomarkers for depression and CVD (i.e., serotonin, vitamin D, lipid profile, and hs‐CRP) in healthy adults who consumed yogurt and engaged in exercise.

## 2 MATERIALS AND METHODS

### 2.1 Participants

The participants were recruited via printed advertisements on notice boards at Gimcheon University. The volunteers who participated in the study were healthy university students aged 20–26 years who were not using any medication and had not been diagnosed with any mental or physical health conditions. All 24 participants provided written informed consent. They were randomly assigned to one of the following four groups of six participants (three men and three women): control (no yogurt or exercise), yogurt (consumption of commercial yogurt twice daily after lunch and dinner), exercise (regular daily exercise), and combination (regular daily exercise and consumption of commercial yogurt twice daily after lunch and dinner). The mean ages of participants in the control, yogurt, exercise, and combination groups were 20.7 ± 0.8, 21.7 ± 2.2, 21.2 ± 1.2, and 21.5 ± 1.6, respectively.

### 2.2 Procedure

Serum from participants’ coagulated blood was used for the assay of serotonin (5‐hydroxytryptamine), vitamin D, and hs‐CRP levels and lipid profile tests. An ethylenediaminetetraacetic acid solution for whole blood was prepared for the assay of hematological parameters. Pre‐ and post‐experimental collection of blood specimens was performed via venipuncture at 8:00 a.m. All participants were advised to fast for 12 hr prior to the specimen collection. At baseline, we performed a serotonin assay using Stat Fax2600 and Spectramax190 (LDN, Nordhorn, Germany). Vitamin D was measured using the ADVIA Centaur XP immunoassay system (SIEMENS, Washington DC, USA). Lipid profile tests (i.e., total cholesterol, LDL‐C, HDL‐C, and TG) and the hs‐CRP assay were performed using a Cobas^®^ 8000 c702 modular analyzer (Roche Diagnostics Limited, Basel, Switzerland). Measurement of hematological parameters, such as white blood cell (WBC), red blood cell (RBC), and platelet count; hematocrit; mean corpuscular volume (MCV); mean corpuscular hemoglobin (MCH); mean corpuscular hemoglobin concentration (MCHC); mean platelet volume (MPV); and hemoglobin, were performed using a HeCo C hematology analyzer (Seac‐Radim, Rome, Italy). In addition, participants’ body weight was measured. All four groups were instructed to maintain their usual food intake for the 2‐week study period. At each meal, the yogurt group consumed 83 g of commercial yogurt (Purmil plain yogurt, Seoul, Korea) in solid form, which was provided to the participants and contained *Lactobacillus acidophilus*,* L bulgaricus*, and *Streptococcus thermophilus*. The exercise group performed regular stretching, 500 jump‐rope exercises, and outdoor walking for 40 min per day. Participants who missed their daily yogurt intake or exercise were required to report this to the researcher immediately. Two weeks later, we performed the assays again and analyzed the changes that had occurred in all four groups.

### 2.3 Statistical analysis

Each group contained six participants. Therefore, we used the Wilcoxon signed‐rank test to analyze nonparametric paired data. GraphPad Prism 5 (GraphPad Software Inc., San Diego, CA, USA) was used to perform the statistical analysis.

To confirm the effects of yogurt and exercise on biomarkers of depressive disorders, we measured changes in serotonin and vitamin D from baseline to the 2‐week assessment (Albert & Benkelfat, 2013; Anglin et al., 2013). CRP is a marker of inflammation and is a useful biomarker for CVD risk (Koh et al., 2005; Soriano‐Guillén et al., 2008); therefore, we assessed changes in hs‐CRP at baseline and 2 weeks later. In addition, to evaluate the effects of yogurt and exercise on diet, we measured participants’ body weight at baseline and 2 weeks later.

## 3 RESULTS

### 3.1 Biomarkers for depressive disorders

As shown in Table 1, serotonin levels increased significantly in the combination group (*p *=* *0.03), and vitamin D levels increased significantly in the exercise group (*p *=* *0.03). Vitamin D levels also increased in the combination group, but the result was nonsignificant (*p *=* *0.31). In addition, all participants were found to be deficient in vitamin D at baseline.

**Table 1:** Changes in serotonin and vitamin D in the yogurt and exercise group (*N *=* *24)

		Control (*n *=* *6)		Yogurt (*n *=* *6)		Exercise (*n *=* *6)		Combination (*n *=* *6)	
		Mean ± *SD*	*p*	Mean ± *SD*	*p*	Mean ± *SD*	*p*	Mean ± *SD*	*p*
Serotonin	Pre	136.48 ± 72.98	0.31	101.07 ± 64.66	0.99	99.78 ± 27.41	0.22	70.08 ± 28.90	0.03
(ng/ml)	Post	118.96 ± 60.78		97.08 ± 52.83		88.28 ± 32.67		84.75 ± 36.32	
Vitamin D	Pre	10.83 ± 3.76	0.69	13.43 ± 6.39	0.44	9.50 ± 5.46	0.03	11,83 ± 6.62	0.31
(ng/ml)	Post	12.90 ± 5.50		15.02 ± 6.92		13.45 ± 3.79		16.47 ± 7.45	

*Notes*

Wilcoxon signed‐rank test.

pre, before study; post, after study; SD, standard deviation.

Significance level: *p *<* *0.05.

### 3.2 Lipid profile

None of the experimental groups showed significant changes in total cholesterol, LDL‐C, or HDL‐C levels during the 2‐week study period. However, TG levels in the combination group decreased significantly (*p = *0.03; Table 2).

**Table 2:** Changes in lipid profiles in the yogurt and exercise groups (*N *=* *24)

		Control (*n *=* *6)		Yogurt (*n *=* *6)		Exercise (*n *=* *6)		Combination (*n *=* *6)	
		Mean ± *SD*	*p*	Mean ± *SD*	*p*	Mean ± *SD*	*p*	Mean ± *SD*	*p*
Total C	Pre	162.17 ± 28.90	0.84	176.00 ± 28.84	0.99	161.33 ± 23.67	0.40	149.80 ± 20.99	0.81
(mg/dl)	Post	163.33 ± 31.82		6.62 ± 0.89		158.00 ± 33.72		153.40 ± 30.44	
HDL‐C	Pre	54.50 ± 8.83	0.84	57.17 ± 10.89	0.17	64.67 ± 9.56	0.67	52.40 ± 8.32	0.13
(mg/dl)	Post	56.33 ± 16.48		62.00 ± 15.74		63.17 ± 11.58		56.20 ± 10.64	
LDL‐C	Pre	97.00 ± 31.40	0.81	110.17 ± 29.45	0.92	85.67 ± 27.55	0.60	85.80 ± 21.58	0.17
(mg/dl)	Post	99.67 ± 29.49		111.67 ± 34.23		87.50 ± 34.05		90.60 ± 25.98	
TG	Pre	97.33 ± 34.88	0.59	82.67 ± 16.52	0.44	90.83 ± 28.71	0.99	109.60 ± 42.13	0.03
(mg/dl)	Post	97.83 ± 35.30		72.33 ± 28.51		94.00 ± 25.71		92.40 ± 42.56	

*Notes*

Wilcoxon signed‐rank test.

pre, before study, post, after study; SD, standard deviation; Total C, total cholesterol; HDL‐C, high‐density lipoprotein cholesterol; LDL‐C, low‐density lipoprotein cholesterol; TG, triglyceride.

Significance level: *p *<* *0.05.

### 3.3 Concentrations of hs‐CRP

The hs‐CRP concentration did not change significantly in the control (from 0.59 ± 0.21 to 0.54 ± 0.19 mg/L, *p *=* *0.31), yogurt (from 0.37 ± 0.14 to 0.74 ± 0.53 mg/L, *p *=* *0.13) or exercise (from 0.46 ± 0.10 to 0.66 ± 0.51 mg/L, *p *=* *0.44) groups during the 2‐week study period; however, the hs‐CRP concentration decreased significantly in the combination group (from 1.24 ± 0.90 to 0.95 ± 0.91 mg/L, *p *=* *0.03).

### 3.4 Hematological parameters

Changes in hematological parameters for the yogurt and exercise groups are shown in Table 3. WBC and RBC did not change significantly in any of the groups. It is interesting that hemoglobin levels decreased significantly in the exercise group (*p *=* *0.04), but no significant change was observed in the combination group. MCH levels and MCHC decreased significantly in both the exercise and combination groups (exercise group: MCH and MCHC: *p*s = 0.03; combination group: MCH: *p *=* *0.03, MCHC: *p *=* *0.04).

**Table 3:** Changes in hematological parameters in the yogurt and exercise groups (*N *=* *24)

		Control (*n *=* *6)		Yogurt (*n *=* *6)		Exercise (*n *=* *6)		Combination (*n *=* *6)	
		Mean ± *SD*	*p*	Mean ± *SD*	*p*	Mean ± *SD*	*p*	Mean ± *SD*	*p*
WBC	Pre	6.13 ± 1.04	0.31	6.23 ± 1.62	0.69	7.29 ± 2.42	0.92	6.95 ± 1.18	0.69
(×10^3^/μl)	Post	6.58 ± 1.74		6.62 ± 0.89		7.43 ± 1.63		7.48 ± 1.22	
RBC	Pre	5.06 ± 0.61	0.44	5.14 ± 0.29	0.44	4.91 ± 0.73	0.22	5.02 ± 0.48	0.31
(×10^6^/μl)	Post	5.09 ± 0.65		4.99 ± 0.54		4.74 ± 0.58		5.11 ± 0.53	
Hgb	Pre	13.43 ± 1.67	0.14	13.67 ± 2.10	0.31	13.77 ± 1.94	0.04	13.93 ± 1.65	0.99
(g/dl)	Post	13.65 ± 1.95		12.97 ± 2.73		12.8 ± 1.64		13.93 ± 1.73	
Hct	Pre	44.27 ± 4.84	0.44	44.22 ± 5.93	0.34	44.00 ± 5.99	0.16	44.22 ± 4.93	0.29
(%)	Post	44.60 ± 5.74		42.78 ± 7.45		42.52 ± 4.69		45.13 ± 5.22	
MCV	Pre	87.50 ± 3.89	0.99	85.67 ± 9.20	0.77	89.83 ± 2.32	0.99	88.17 ± 2.48	0.99
(fL)	Post	87.50 ± 4.42		85.50 ± 9.52		89.83 ± 2.14		88.33 ± 2.42	
MCH	Pre	27.24 ± 2.05	0.89	26.36 ± 3.49	0.09	28.08 ± 0.75	0.03	27.70 ± 1.06	0.03
(pg)	Post	27.06 ± 1.41		25.74 ± 3.77		26.94 ± 0.92		27.20 ± 1.01	
MCHC	Pre	30.33 ± 1.18	0.79	30.83 ± 0.97	0.16	31.35 ± 0.26	0.03	31.48 ± 0.61	0.04
(g/dl)	Post	30.55 ± 0.56		30.12 ± 1.29		30.02 ± 1.13		30.88 ± 0.51	
Plt	Pre	289 ± 43	0.63	275 ± 60	0.03	265 ± 28	0.99	245 ± 42	0.99
(×10^3^/μl)	Post	281 ± 51		253 ± 60		278 ± 59		247 ± 20	
MPV	Pre	11.02 ± 0.52	0.31	10.92 ± 0.99	0.06	11.02 ± 0.66	0.34	10.77 ± 0.67	0.17
(fL)	Post	11.17 ± 0.46		10.58 ± 0.87		10.93 ± 0.67		10.95 ± 0.67	

*Notes*

Wilcoxon signed‐rank test.

pre, before study; post, after study; SD, standard deviation; WBC, white blood cell; RBC, red blood cell; Hgb, hemoglobin; Hct, hematocrit; MCV, mean corpuscular volume; MCH, mean corpuscular hemoglobin; Plt, platelet; MPV, mean platelet volume. Significance level: *p*<0.05.

### 3.5 Body weight

The participants in the control (61.48 ± 17.60 kg to 61.05 ± 17.90 kg, *p *=* *0.56), yogurt (57.00 ± 7.29 kg to 57.75 ± 9.90 kg, *p *=* *0.99) and exercise (66.67 ± 13.91 kg to 66.77 ± 14.17 kg, *p *=* *0.81) groups did not show marked changes in body weight. The participants in the combination group showed an average weight loss of approximately 1 kg (70.40 ± 15.92 kg to 69.34 ± 16.19 kg); however, the result was nonsignificant (*p *=* *0.10).

## 4 DISCUSSION

In the current study, the significance of the results regarding the prevention of depression and CVD observed for the combination group was stronger relative to that of the results for the yogurt and exercise groups separately.

We included serotonin and vitamin D as biomarkers for depression. Serotonin levels did not change in the separate yogurt and exercise groups but increased significantly in the combination group, despite the short study period and small sample size relative to those of previous studies. Serotonin is synthesized in the digestive tract and central nervous system (CNS), with almost 90% synthesized by enterochromaffin cells in the gastrointestinal epithelium (Jenkins, Nguyen, Polglaze, & Bertrand, 2016; Mawe & Hoffman, 2013; Mazák, Dóczy, Kökösi, & Noszál, 2009). Recent studies reported that the gut microbiota was associated with serotonin synthesis and affected the CNS via the gut˗brain axis, and serotonin acts as a neurotransmitter at these terminals. In a germ‐free animal model, levels of circulating tryptophan, a precursor of serotonin, were higher relative to those in a conventionally colonized control, and colonization of a germ‐free animal restored tryptophan levels, controlled anxious behavior, and increased plasma serotonin levels, suggesting that the gut microbiota played an important role in tryptophan metabolism and CNS neurotransmission (Clarke et al., 2013; Wikoff et al., 2009). Recent studies reported that probiotics were associated with the pathogenesis of depression and could play an important role in reducing the risk of depression (Huang et al., 2016). Based on these results, probiotics could alleviate depressive symptoms by activating tryptophan metabolism in the serotonergic system. Exercise is also known to exert a beneficial effect on mood and physical health. Moreover, it also reduces depressive symptoms and should be considered as a prospective adjunctive treatment for mood disorders (Hearing et al., 2016). Soares, Naffah‐Mazzacoratti, and Cavalheiro (1994) found that serum serotonin levels in physically trained participants were higher relative to that observed in untrained participants. In the current study, the increase in levels of vitamin D, another biomarker for depression, in the exercise group was significantly greater relative that observed in the yogurt group, indicating that exercise could improve depression by increasing vitamin D levels. Solar UV‐B irradiation is a major natural source of vitamin D (Holick, 2006), and the exercise group performed exercises in outdoor settings and was therefore exposed to higher levels of solar UV‐B irradiation, relative to the yogurt group, which could also have contributed to the increase in vitamin D levels. It is interesting that the participants were healthy young adults, and they all exhibited vitamin D deficiency at baseline.

In addition, despite the short duration of the study period, yogurt and exercise combined, but not separately, increased serotonin production. These results suggest that exercise could trigger tryptophan metabolism by probiotics and increase serotonin production. Therefore, yogurt intake combined with exercise could be more effective in preventing depression, relative to either yogurt or exercise alone.

Furthermore, we measured the lipid profile and hs‐CRP in each group, to examine the beneficial effects of the combination of yogurt and exercise on CVD. The results showed that the combination of yogurt and exercise decreased TG and hs‐CRP levels. Lipids in the blood are classified as cholesterol and TG. Cholesterol is transported inside lipoproteins, of which there are several types including chylomicron, very low‐density lipoprotein, LDL, and HDL (Rohilla et al., 2012). Several studies have reported that yogurt consumption or engagement in exercise exerted a preventive effect on CVD (Astrup, 2014; Ataie‐Jafari, Larijani, Alavi Majd, & Tahbaz, 2009; Villella & Villella, 2014; Wikoff et al., 2009). In particular, probiotic yogurt or exercise reduced the risk of CVD by reducing total serum cholesterol levels or obesity. In hypercholesterolemic individuals, consumption of probiotic yogurt has been shown to reduce total serum cholesterol, indicating that probiotics are involved in lipid metabolism (Ataie‐Jafari et al., 2009). The results also showed that probiotic yogurt reduced hs‐CRP levels, which is consistent with the results of a study in which consumption of probiotic yogurt for 9 weeks reduced hs‐CRP levels in pregnant women (Asemi et al., 2011).

The current results also showed that TG and hs‐CRP levels decreased significantly in the combination group. TG levels also decreased in the yogurt group, but the result was nonsignificant. These results indicate that yogurt could play a role in reducing CVD risk by decreasing TG and hs‐CRP levels, and this effect could be augmented by exercise.

Hematological parameters, including WBC, RBC, platelet count, hematocrit, MCV, MCH, MCHC, MPV, and hemoglobin, are affected by pathological and physiological conditions (Rodak, Frisma, & Keohane, 2012). In addition, exercise can induce the release of platelets from the spleen and increase the circulating platelet count (Heber & Volf, 2015). It is interesting that in the exercise group, hemoglobin, MCH, and MCHC decreased significantly. This result appeared to indicate sports anemia. Radomski, Sabiston, and Isoard (1980) reported that hypochromic premature RBCs are released from the bone marrow in sustained submaximal exercise, which results in the reduction in hemoglobin. However, the consumption of yogurt combined with exercise suppressed the reduction in hemoglobin, MCH, and MCHC, as hemoglobin levels did not decrease and reductions in MCH and MCHC levels were smaller relative to those observed in the exercise group. These results imply that yogurt could contribute to the prevention of anemia resulting from exercise. Few studies have examined the combination of yogurt and exercise. In a study examining the effects of yogurt on hemoglobin, Mohammad, Molloy, Scott, and Hussein (2006) reported that the consumption of yogurt containing *Lactobacillus acidophilus* increased hemoglobin levels; however, in the current study, hemoglobin levels did not increase in the yogurt group. This discrepancy could have occurred because of differences in sample size, participants’ age, and the study duration between the two studies. Mohammad et al.'s (2006) study included 12 participants aged 11 years, and the duration of their study period was 42 days. Based on these findings, although yogurt could relieve anemia resulting from exercise, via the regulation of hemoglobin, the effect of yogurt on hemoglobin levels requires further examination in studies with large populations and longer study periods, as there is a lack of previous research.

This study was subject to some limitations; for example, the study period was too short, and the sample size was small. Despite these limitations, the study provided some significant results. However, further studies with larger sample sizes and a greater variety of biomarkers are required to allow generalization of the combined effects of yogurt and exercise on depression and CVD.

In conclusion, the combination of yogurt and exercise could reduce the risk of depression by increasing serotonin. In addition, it could prevent CVD via reductions in TG and hs‐CRP levels. The results of this study indicated that the combination of yogurt and exercise was more effective, relative to yogurt or exercise alone, in preventing depression and CVD. Furthermore, the study findings could be helpful for people who wish to maintain good mental and physical health, and provide a foundation for instructions in this regard for patients with depression or CVD.

## CONFLICT OF INTEREST

The authors declare that they do not have any conflict of interest.

## ETHICAL STATEMENTS

Ethical Review: This study was not approved by the internal review board of our institute. Because this study was carried out before our institute was established. However, this study was designed according to the declaration of Helsinki: Each participant signed a consent form from confirming their agreement to participate in this study.

Informed Consent: Written informed consent was obtained from all study participants.
